# Qualitative and Quantitative Analysis of *Andrographis paniculata* by Rapid Resolution Liquid Chromatography/Time-of-Flight Mass Spectrometry

**DOI:** 10.3390/molecules181012192

**Published:** 2013-09-30

**Authors:** Yong-Xi Song, Shi-Ping Liu, Zhao Jin, Jian-Fei Qin, Zhi-Yuan Jiang

**Affiliations:** 1 Department of Pharmacy, The First Affiliated Hospital of Harbin Medical University, Harbin 150001, China; E-Mails: shipingliu@sina.com (S.-P.L.); shipingliu666@126.com (Z.J.); 2 Harbin Sanlian Pharmaceutical Co., LTD, Harbin 150025, China; 3 Heilongjiang Province Agricultural Reclamation Administration of the Centers for Disease Control and Prevention, Harbin 150090, China; E-Mail: zhongyao150001@126.com

**Keywords:** *Andrographis paniculata*, rapid resolution liquid chromatography, time-of-flight tandem mass, diterpenoid lactones

## Abstract

A rapid resolution liquid chromatography/time-of-flight tandem mass spectrometry (RRLC-TOF/MS) method was developed for qualitative and quantitative analysis of the major chemical constituents in *Andrographis paniculata*. Fifteen compounds, including flavonoids and diterpenoid lactones, were unambiguously or tentatively identified in 10 min by comparing their retention times and accurate masses with standards or literature data. The characteristic fragmentation patterns of flavonoids and diterpenoid lactones were summarized, and the structures of the unknown compounds were predicted. Andrographolide, dehydroandrographolide and neoandrographolide were further quantified as marker substances. It was found that the calibration curves for all analytes showed good linearity (*R*^2^ > 0.9995) within the test ranges. The overall limits of detection (LODs) and limits of quantification (LOQs) were 0.02 μg/mL to 0.06 μg/mL and 0.06 μg/mL to 0.2 μg/mL, respectively. The relative standard deviations (RSDs) for intra- and inter-day precisions were below 3.3% and 4.2%, respectively. The mean recovery rates ranged from 96.7% to 104.5% with the relative standard deviations (RSDs) less than 2.72%. It is concluded that RRLC-TOF/MS is powerful and practical in qualitative and quantitative analysis of complex plant samples due to time savings, sensitivity, precision, accuracy and lowering solvent consumption.

## 1. Introduction

*Andrographis paniculata* (Burm.f.) Nees (Acanthaceae) known as Chuan-xin-lian in Chinese, is widely used in China, India, and other Southeast Asian countries [1]. *A. paniculata* has also been recorded in the Chinese Pharmacopoeia for a long time [2]. It possesses various pharmacological effects for the treatment of the common cold, fever and non-infectious diarrhea [3,4]. Many Chinese medical preparations containing *A. paniculata* or its extract are listed in Chinese Pharmacopoeia, such as “Chuanxinlian tablet”, “Andrographolide drop pill”, “Chuanxinlian capsule”, *et al.* [2]. The main active constituents of *A. paniculata* include flavonoids and diterpenes, especially labdane diterpenes such as andrographolide, dehydroandrographolide and neoandrographolide [5,6,7,8,9]. A number of pharmacological activities of these marker components were previously reported [10,11,12,13,14,15,16].

Several chromatographic methods have been reported for analysis of these diterpenoids, including thin-layer chromatography, high performance liquid chromatography, and capillary electrophoresis. However, most of them could only determine two or three constituents [17,18,19,20,21,22,23,24]. Moreover, these methods seemed to be associated with some disadvantages such as complicated sample preparation, low sensitivity and time-consuming. Most recently, a quantitative proton nuclear magnetic resonance technique has been successfully applied to quantify andrographolide, dehydroandrographolide, deoxyandrographolide and neoandrographolide in *A. paniculata* [25]. However, nuclear magnetic resonance instruments are still inaccessible for most laboratories worldwide.

In this study, a rapid resolution liquid chromatography/time-of-flight tandem mass spectrometry (RRLC-TOF) was developed to identify and quantify the major constituents of *A. paniculata*. From the point of view of reducing the consumption of solvent and time savings, RRLC–TOF/MS may be a quite useful technique to identify and determine active constituents in Traditional Chinese Medicines, becoming a valuable addition to the existing analysis tools.

## 2. Results and Discussion

### 2.1. Optimization of Sample Preparation and Chromatographic Conditions

**Figure 1:**
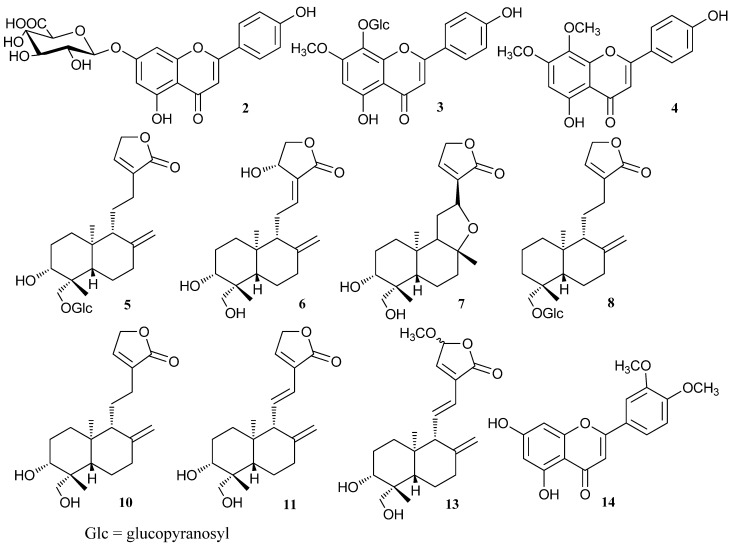
The structures of compounds identified in *Andrographis paniculata.*

**Figure 2:**
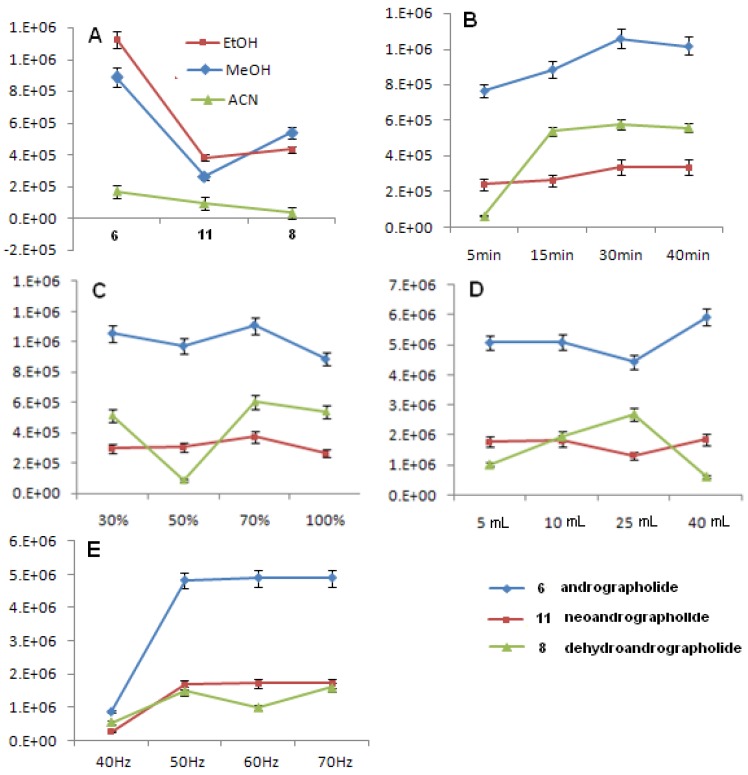
Effects of the types of extraction solvent (**A**), time (**B**), concentration of solvents (**C**), ratio of liquid to solid (**D**) and ultrasonic frequency (**E**) were investigated on yields of andrographolide (**6**), neoandrographolide (**8**) and dehydroandrographolide (**11**) in *A. paniculata*.

Andrographolide (**6**), dehydroandrographolide (**8**) and neoandrographolide (**11**) are selected as marker compounds for investigating the extract yields by a common HPLC-UV method. Their chemical structures can be seen in Figure 1. The extraction solvent plays a critical role in obtaining a satisfactory extraction of compounds from plant samples and in obtaining a good chromatographic fingerprint representing the quality control of a herbal medicine. In this study, ethanol, methanol and acetonitrile were used as extraction solvents. As shown in Figure 2A, except for compound **8**, ethanol exhibits relatively higher extraction efficiency for compounds **6** and **11** as compared to methanol and acetonitrile. Thus, considering safety and economy, ethanol was applied for the subsequent experiments.

Extraction time had a close relationship with extraction efficiency. In the assay, extraction efficiency in samples (0.2 g) was compared by sonication with 10 mL of ethanol at 60 kHz for 5, 15, 30 and 40 min, respectively. The results indicated that the highest extraction efficiency was obtained by sonication for 30 min in pure ethanol (Figure 2B). In this study, effects of the ethanol concentration (30%; 50%; 70%; 100%) and volume (5; 10; 25; 40 mL) on the extracting yield were also investigated. By comparing peak areas of the three markers, it was found that, when 70% ethanol was employed, the peak areas of the three investigated components reached the highest values (Figure 2C). Generally, usage of larger volume of extracting solvent could obtain larger amount of extract [26]. Sometimes, the large ratio of liquid to material also resulted in the decrease of extraction yield [27]. In this study, the significant decrease of yield of **8** was observed when 40 mL of 70% ethanol was used. However, when 25 mL of 70% ethanol was applied, the yield of **6** and **11** was obviously decreasing. Thus, 10 mL of 70% ethanol may be a better choice to compromise (Figure 2D). Finally, effects of sonication frequency (40, 50, 60 and 70 kHz) on the extracting yield were investigated. The results indicated that the highest extraction efficiency was obtained by sonication at 50 kHz (Figure 2E).

From the above experiments, it was demonstrated that the most suitable ultrasonic extraction condition for the three markers from *Andrographis paniculata* was 0.2 g plant sample with 10 mL of 70% ethanol and extraction for 30 min at under 50 kHz ultrasonic irradiation.

**Figure 3:**
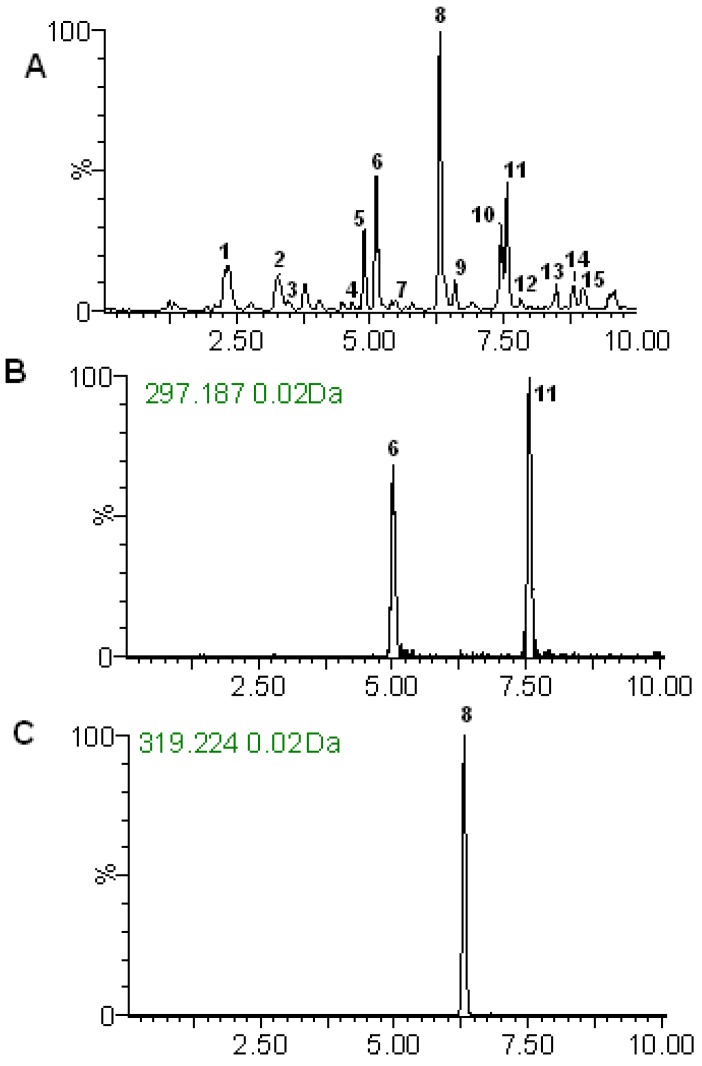
(**A**) Total ion chromatogram in positive ion mode of *A. paniculata*; (**B**) The narrow widow extracted ion chromatograms (nwXICs) chromatograms for *m/z* 297.187 (**6** and **11**); (**C**) The nwXICs chromatograms for *m/z* 319.224 (**8**). The retention time is defined as the minute.

By testing different types of chromatographic columns it was determined that an ACE C18 column is suitable for separating the bioactive constituents in *A. paniculata*. The selection of mobile phase played an important role in achieving good chromatographic separation and appropriate ionization. The effect of different mobile phase compositions on chromatographic separation was compared. Acetonitrile-water possessed higher resolution and better peak shape than methanol-water. Several mobile phase additives, such as formic acid, acetic acid and ammonium acetate, were used to achieve better resolution of the analytes. It was found that the good signal intensity, resolution and peak shape were achieved when formic acid was added to both acetonitrile and aqueous solution. But with the adding amount of formic acid increasing, the signal intensity had the downward trend. Ultimately, 0.1% formic acid added to the mobile phase was suitable. There was no sharp effect upon changing the column temperature (25–35 °C) on either the peak symmetry or the resolution of the eluted peaks, but only a marked increasing in the retention time and so the column temperature of 35 °C was used. The effect of different flow rates (0.2–0.4 mL/min) has been also examined. There was no obvious tailing and poor peaks symmetry appeared at lower values of the flow rate, so 0.2 mL/min was selected as the optimum flow rate. Under these optimum conditions, all the studied chemical constituents were well separated from each other by RRLC-TOF/MS method (Figure 3A).

### 2.2. RRLC-TOF Analysis

#### 2.2.1. Identification of Diterpene Lactones

**Figure 4:**
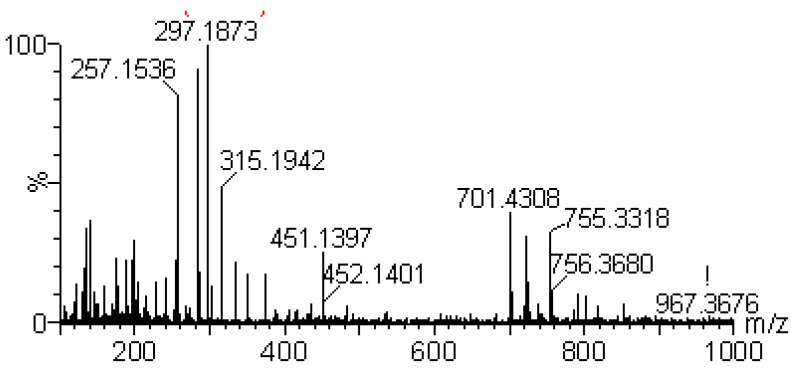
ESI (+) MS spectrum of compound **6**.

**Scheme 1:**
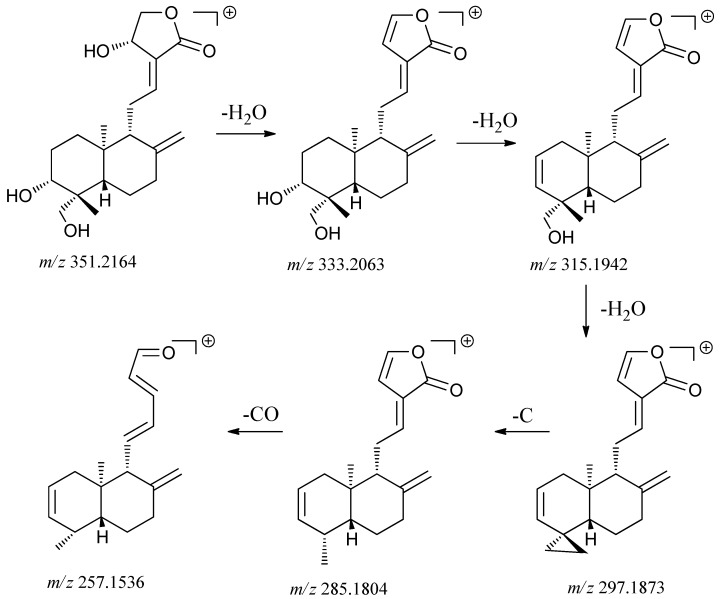
The proposed fragmentations pathways of andrographolide (**6**).

Ten diterpene lactones were detected in *A. paniculata* and their structures are shown in Figure 1. These compounds were classified into monomers and polymers of diterpene lactones. The positive ion mode was much more useful for the analysis of these kinds of compounds. Compounds **5**–**11** and **13** are monomers with multiple hydroxyls group in their structures whose characteristic fragmentation behavior was the successive loss of one, two and multiple H_2_O molecules [28,29]. Compounds **5** and **8** have glucosyl groups attached to the core molecules by glycosidic linkages which were readily dissociated, so their characteristic fragment ions were formed by the losses of a glucosyl group (162 Da). Figure 4 is mass spectrum of compound **6**, which is a typical monomer. A plausible fragmentation pathway of **6** is shown in Scheme 1.

On the basis of above analysis, compounds **6**, **8** and **11** were unambiguously identified as andrographolide, neoandrographolide and dehydroandrographolide by comparison with authentic references under the same chromatography conditions. Compounds **5**, **7**, **10** and **13** were tentatively identified as 14-deoxyandrographiside, isoandrographolide, 14-deoxyandrographolide and 3,19-dihydroxy-15-methoxy-ent-labda-8(17),11,13-trien-16,15-olide, respectively, by exact molecular formulae matching, fragmentation information and retention behaviors as well as the literature data [6,8,9]. However, compound **9** was an unreported natural product, tentatively identified as hydroxylated compound **13** by comparison of the exact molecular formulae with compound **13** and their fragmentation information [9]. However, it is difficult to determine the position of hydroxylation by the single mass method. Both **12** and **15**, bis-andrographolide compounds, are unreported natural products, which are different from the reported bis-andrographolides A–D in *A. paniculata* [30,31]. Taking compound **12** as example, it showed a molecular ion at *m/z* [M+H]^+^ 681.3962 and with successive loss of four molecules of water followed by a fragment ion at *m/z* 663.3945, 645.3752, 627.3672 and 609.3548. The further fragment ion at *m/z* 297.1833 supports the proposed the hypothesis that compound **12** should be a bis-andrographolide compound.

#### 2.2.2. Identification of Flavone Compounds

**Scheme 2:**
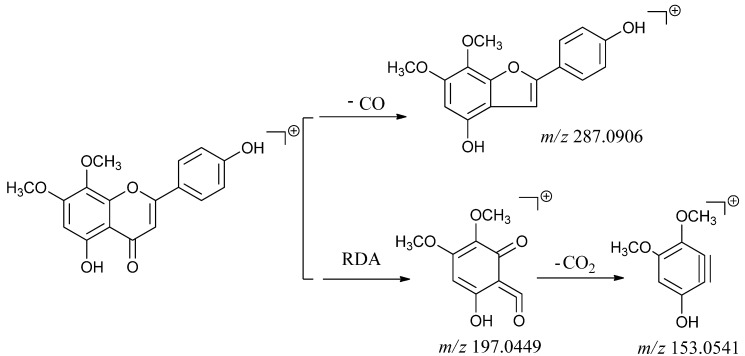
The proposed fragmentations pathways of compound **4**.

The characteristic fragmentation behavior of the flavone aglycones (compounds **4** and **14**) were loss of CO (28 Da) and a reverse Diels-Alder (RDA) reaction [32,33]. The peak of [M+H-2CH_3_]^+^ at suggested two OCH_3_ substitutions on compounds **4** and **14**. In the MS/MS spectra of compound **4**, the fragment ions with the highest abundance at *m/z* 287.0906, 197.0449 and 153.0541 (Table 1), suggesting the 5-hydroxy-7,8-dimethoxy-substitution may be located in the flavone A ring, so compound **4** was tentatively identified as 5,4'-dihydroxy-7,8-dimethoxyflavone by the exact molecular formulae matching and fragmentation information retention behaviors. The possible fragmentation pathway of compound **4** is summarized in Scheme 2. Meanwhile, compound **14** showed similar fragmentation and had the same molecule weight with compound **4**, so it was tentatively identified as an isomer of compound **14**.

**Table 1:** Quasi-molecular ions of compounds in *A. paniculata* sample by RRLC-TOF analysis.

No.	t_R_ (min)	MS and MS/MS (*m*/*z*)	Type	Identification
**1**	2.33	[M+H]^+^ 463.3319	Flavone	Unknown
		[M+H-H_2_O]^+^ 445.3206		
		[Aglycone+H]^+^ 287.0592		
**2**	3.28	[M+H]^+^ 447.0887	Flavone	Apigenin-7-O-β-D-glucuronide
		Calculated [M+H]^+^ 447.0927		
		[2M+H]^+^893.1624		
		[Aglycone+H]^+^ 271.0568		
**3**	3.49	[M+H]^+^463.1211	Flavone	(5,4'-dihydroxy-7-methoxy-8-O-*β*-d-glucopyrarosyl-flavone
		Calculated [M+H]^+^ 463.1240		
		[M+H-H_2_O]^+^445.3185		
		[Aglycone+H]^+^ 301.0673		
**4**	4.67	[M+H]^+^ 315.0859	Flavone	5,4'-dihydroxy-7,8-dimethoxy-flavone
		Calculated [M+H]^+^ 315.0869		
		[M+H-CO]^+^ 287.1990		
		[M+H-2CH_3_]^+^285.1879		
		[M+H-CH_3_]^+^ 300.0630		
		Typical fragment 287.2906		
		Typical fragment 197.0449		
		Typical fragment 153.0941		
**5**	4.89	[M+H]^+^ 497.2740	Diterpene	14-deoxyandrographiside
		Calculated [M+H]^+^ 497.2751		
		[M+H-Glc]^+^ 335.2223		
		[M+H-Glc-H_2_O]^+^ 317.2087		
		[M+H-Glc-2H_2_O]^+^299.1959		
		Typical fragment 287.1954		
		Typical fragment 259.1660		
		[M+Na]^+^ 519.2543		
		[2M+H]^+^ 993.5358		
**6**	5.11	[M+H]^+^351.2164	Diterpene	Andrographolide
		Calculated [M+H]^+^ 351.2171		
		[M+H-H_2_O]^+^ 333.2063		
		[M+H-2H_2_O]^+^ 315.1929		
		[M+H-3H_2_O]^+^ 297.1798		
		Typical fragment 285.1804		
		Typical fragment 257.1501		
		[2M+H]^+^ 701.4224		
**7**	5.40	[M+H]^+^351.2173	Diterpene	Isoandrographolide
		Calculated [M+H]^+^ 351.2171		
		[M+H-H_2_O]^+^ 333.2065		
		[M+H-2H_2_O]^+^ 315.1954		
		[M+H-3H_2_O]^+^ 297.1828		
		Typical fragment 285.1847		
		Typical fragment 257.1558		
		[2M+H]^+^ 701.4210		
**8**	6.30	[M+H]^+^ 481.2825	Diterpene	Neoandrographolide
		Calculated [M+H]^+^ 481.2801		
		[M+H-Glc]^+^ 319.2288		
		[M+H-Glc-H_2_O]^+^ 301.2125		
		Typical fragment 289.2166		
		Typical fragment 261.1866		
		[M+Na]^+^ 503.2672		
		[M+NH_4_]^+^ 498.3100		
		[2M+H]^+^ 961.5472		
**9**	6.60	[M+H]^+^ 379.2119	Diterpene	Unknown
		[M+Na]^+^ 401.1998		
		[M+K]^+^ 417.1768		
		[M+H-H_2_O]^+^ 361.2029		
		[M+H-2H_2_O]^+^ 343.1904		
		[M+H-2H_2_O-CH_2_]^+^ 329.1776		
		[M+H-3H_2_O-CH_2_]^+^ 311.1636		
		Typical fragment 283.1701		
		Typical fragment 257.1414		
		[2M+H]^+^ 757.4134		
**10**	7.44	[M+H]^+^ 335.2202	Diterpene	14-deoxyandrographolide
		Calculated [M+H]^+^ 335.2222		
		[M+Na]^+^ 357.2104		
		[M+H-H_2_O]^+^ 317.2092		
		[M+H-2H_2_O]^+^ 299.1965		
		Typical fragment 287.1896		
		Typical fragment 259.1652		
		[2M+H]^+^ 669.4372		
**11**	7.57	[M+H]^+^ 333.2075	Diterpene	Dehydroandrographolide
		Calculated [M+H]^+^ 333.2066		
		[M+H-H_2_O]^+^ 315.1942		
		[M+H-2H_2_O]^+^ 297.1787		
		Typical fragment 285.1805		
		Typical fragment 257.1506		
		[2M+H]^+^ 665.4048		
**12**	7.83	[M+H]^+^ 681.3962	Diterpene	Unknown©
		[M+Na]^+^ 703.3758		
		[M+H- H_2_O]^+^ 663.3945		
		[M+H-2H_2_O]^+^ 645.3752		
		[M+H-3H_2_O]^+^ 627.3672		
		[M+H-4H_2_O]^+^ 609.3548		
		Typical fragment 297.1833		
		Typical fragment 269.1812		
**13**	8.48	[M+H]^+^ 363.2169	Diterpene	3,19-Dihydroxy-15-methoxy-ent-labda-8(17),11,13-trien-16,15-olide
		Calculated [M+H]^+^ 363.2171		
		[M+Na]^+^ 385.1991		
		[M+H- H_2_O]^+^ 345.2104		
		[M+H-2H_2_O]^+^ 327.1988		
		[M+H-2H_2_O-CH_2_]^+^ 313.1779		
		[M+H-3H_2_O-CH_2_]^+^ 295.1685		
		Typical fragment 283.1694		
		Typical fragment 255.1414		
		[2M+H]^+^ 725.4254		
**14**	8.80	[M+H]^+^315.0829	Flavone	Dihydroxydimethoxyflavone
		Calculated [M+H]^+^ 315.0869		
		[M+H-CO]^+^ 287.1985		
		[M+H-CH_3_]^+^ 300.0635		
		Typical fragment 271.0628		
		Typical fragment 197.0524		
**15**	8.98	[M+H]^+^ 697.4302	Diterpene	Unknown
		[M+Na]^+^ 719.4150		
		[M+H-3H_2_O-CH_2_]^+^ 629.3845		
		[M+H-4H_2_O-CH_2_]^+^ 611.3734		
		[M+H-4H_2_O-CH_2_-C]^+^ 599.3704		
		Typical fragment 297.1829		
		Typical fragment 255.1491		

Compounds **2** and **3** have similar fragmentation pathways to produce the diagnostic aglycone fragment in positive ion mode, suggesting these compounds are O-glycosyl flavonoids. In the positive MS/MS spectra, the fragment ions with the highest abundance at *m/z* 271.0568 and 301.0673 (Table 1) suggest that their aglycone may be luteolin, 5,8,4'-trihydroxy-7-methoxyflavone, on the basis of the structures of O-glycosyl flavonoids that have been reported before from *A. paniculata* [6]. The structure of the sugar residue could be determined by calculating the difference between the molecular mass and the aglycone mass.

### 2.3. Methodological Validation of the Quantitative Analysis

LC-TOF instruments have been successfully applied to quantify some active molecules in many fields. Ions chromatograms can be extracted using a narrow mass window. This increases the possibility that the chromatographic peaks are free from background or co-elutes interference. The three components were analyzed by MS in ESI positive ion mode. It can be seen from Figure 3A that all targeted compounds andrographolide (**6**), neoandrographolide (**8**) and dehydroandrographolide (**11**) were baseline separated within 10 min in standard and samples, which represented an approximate five-fold reduction in the analysis time in comparison to published HPLC methods [34,35].

Quantitation was carried out working in the V mode using the narrow widow extracted ion chromatograms (nwXICs) of each compound (extracted using 20 mDa window, Figure 3B,C). In ESI positive ion of V mode with a sample cone at 40 V, [M+H-3H_2_O]^+^, [M+H-Glc]^+^ and [M+H-2H_2_O]^+^ (297.1873, 319.2267 and 297.1873) for compounds **6**, **8** and **11** were considered better stability and higher abundance. Therefore, the narrow widow extracted ions 297.1873 and 319.2267 were chosen for quantitation for compounds **6**, **8** and **11** (Figure 3B,C**)**. The analytes were identified by comparing their retention times and accurate *m/z* data with standard solutions containing the corresponding compounds.

It is well-known that the occurrence of matrix effects in LC-MS has an important impact on the quantitation of the constituents in complex plant samples. Matrix effects can influence the response when compared to standards in neat solvents. In this work, the *A. paniculata* samples and samples added with different concentrations of standard solutions were analyzed using LC-TOF/MS, and the result was compared to standards in solvent (methanol without matrix). Relative recoveries were used to determine the matrix effects: relative recoveries = (sample contents with adding–original contents)/contents of standard solutions for adding. The relative recoveries for all three compounds ranged between 93.7% and 112%, thus showing minimal matrix suppression or enhancement of this method.

Table 2 provides the validation parameters including calibration curves, linearity ranges, LODs, and LOQs. As can be seen, linearity of analytical response is excellent with correlation coefficients higher than 0.9995 offering a linear dynamic range of about two orders of magnitude, which is enough to promise successful quantitative applications in routine analysis and quality control of *A. paniculata.* LODs of three compounds varied from 0.02 μg/mL to 0.06 μg/mL, and LOQs ranged from 0.06 μg/mL to 0.2 μg/mL.

**Table 2:** Calibration parameters of UPLC-MS analysis for the three compounds.

No.	Regression equation	Linear range (μg/mL)	*R*^2^	LODs (μg/mL)	LOQs (μg/mL)
**6**	y = 22.35x − 9.56	0.2–100	0.9996	0.06	0.2
**8**	y = 46.00x − 18.88	0.2–100	0.9995	0.02	0.06
**11**	y = 13.25x + 5.813	1.0–100	0.9998	0.06	0.2

The intra-day and inter-day precisions were investigated by determining the three constituents in a *A. paniculata* sample six times per day and on three consecutive days. The intra-day and inter-day precisions of the determination of the three constituents were less than 3.3% and 4.2%, respectively.

The relative recovery experiment results showed acceptable losses in pretreatment procedure of *A. paniculata* and its preparations in three different levels with recoveries ranged from 96.7% to 104.5% with the relative standard deviations (RSDs) less than 2.72%.

The developed fast LC-TOF/MS method was then applied to analysis of three analytes in 10 samples, *i.e.*, four raw materials of *A. paniculata*, three *Andrographis* tables and three *Andrographis* extracts. The analytical contents were summarized in Table 3. The results demonstrate a successful application of this fast RRLC-ESI-TOF/MS assay for the quantification of the major active compounds in different *A. paniculata* samples and preparations.

**Table 3:** Description of the test samples.

No.	Source	Geographical regions	Comp. 6 ^a^	Comp. 8 ^b^	Comp. 11 ^c^
1	*A. paniculata*	Unknown	0.23	0.27	0.07
2	*A. paniculata*	Guangxi	0.65	0.73	0.80
3	*A. paniculata*	Jiangxi	0.24	0.04	0.09
4	*A. paniculata*	Unknown	1.08	0.16	0.25
5	Andrographis Extract	Sichuan	6.73	11.95	10.75
6	Andrographis Extract	Sichuan	3.56	9.28	16.54
7	Andrographis Extract	Sichuan	7.24	9.68	17.45
8	Andrographis Tablet	Guangxi Fanglue	1.35	0.83	0.67
9	Andrographis Tablet	Heilongjiang Wusulijiang	0.33	0.42	0.52
10	Andrographis Tablet	Guangdong Boluoxianfeng	1.61	0.63	0.69

^a^ Comp. **6**, andrographolide; ^b^ Comp. **8**, neoandrographolide; ^c^ Comp. **11**, dehydroandrographolide. *A. paniculata* and Andrographis extracts are expressed as g/g × 100%, while Andrographis tablet are expressed as g/tablet × 100%.

## 3. Experimental

### 3.1. Chemicals and Reagents

Chromatography grade methanol was purchased from Merck (Darmstadt, Germany). Deionized water was purified by Milli-Q system (Millipore, Bedford, MA, USA). *A. paniculata* and its commercial samples can be seen in Table 3. Standards of andrographolide, dehydroandrographolide and neoandrographolide were all ordered from the Chinese National Institute of Control of Pharmaceutical and Biological Products (Beijing, China). Their structures can be seen in Figure 1. All other reagents were of analytical grade.

### 3.2. Analytical Sample Preparation

The dried powders of *A. paniculata* (0.2 g, 40 mesh) were accurately weighed and extracted for 30 min under 50 Hz ultrasound with 10 mL of 70% ethanol solution. Then the resultant mixture was adjusted to the original weight and the supernatant were filtered through a 0.22 μm membrane before RRLC injection. Meanwhile, the sugar-coating of 10 Andrographis tablets was removed and they were porphyrized in a porcelain mortar. The Andrographis tablet powders were extracted under ultrasound with 10 mL of 70% ethanol solution for 30 min. Then the resultant mixture was adjusted to the original weight and the supernatant were filtered through 0.22 μm membrane before RRLC injection. The dried powders of Andrographis extract (1.0 g) were accurately weighed and dissolved in a 25 mL volumetric flask with of 70% ethanol. The ethanol solution was filtered through a 0.22 μm filter membrane for RRLC injection.

### 3.3. RRLC-TOF Apparatus and Conditions

The RRLC-MS analysis was performed on a LC2010 HPLC system (Shimadzu, Kyoto, Japan) coupled with a Waters Premier MS equipped with electrospray ionization. For the reversed-phase RRLC analysis, an ACE Excel 3 Super C18 column (100 mm × 2.1 mm i.d., 3.0 μm) was used. The column temperature and sample temperature were maintained at 35 °C and 4 °C, respectively; the flow rate of the mobile phase was 0.20 mL/min; the injection volume was fixed at 2.0 μL. Mobile phase A consisted of 0.1% formic acid in acetonitrile, while mobile phase B consisted of 0.1% formic acid in water. The column was eluted with a linear gradient of 30%–40% A over initial to 2.0 min, 40%–75% A over 2.0–8.0 min, 75%–100% A over 8.0–9.0 min, 100%–30% A over 9.0–9.5 min, 30% A over 9.5–15.0 min.

The mass spectrometric full-scan data were acquired in the positive ion by V mode from 100 to 1,000 Da with a 0.1 s scan time. Other conditions were as follows: capillary voltage of 4.0 kV, desolvation temperature of 350 °C, sample cone voltage of 40 V, extraction cone voltage of 3.0 V, source temperature of 100 °C, cone gas flow of 50 L/h and desolvation gas flow of 400 L/h for negative ion mode. Data were centroided and mass was corrected during acquisition using an external reference (Lock-Spray^TM^) consisting of a 0.2 ug/mL solution of leucine enkephalin infused at a flow rate of 1.0 μL/min via a lockspray interface, generating a reference ion for positive ion mode ([M+H]^+^ = 556.2771) to ensure accuracy during the MS analysis.

### 3.4. Standard Preparation and Calibration Curves

An ethanol stock solution containing all three reference standards was prepared by dissolving the reference standards in ethanol to a final concentration of 500 μg/mL for each reference standard, then diluted the mixture stock solution to appropriate concentration to establish calibration curves. Each calibration curve concentration was performed in triplicate. All calibration curves were constructed from peak areas of reference standards versus their concentrations. The lowest concentration of working solution was diluted with ethanol to yield a series of appropriate concentrations, and the LOD and LOQ under the chromatographic conditions were separately determined at an S/N of 3 and 10, respectively. The results were listed in Table 2.

## 4. Conclusions

The chemical fingerprint profile of *A. paniculata* was systematically investigated by the RRLC-TOF/MS technique. A total of fifteen compounds were identified or tentatively characterized in *A. paniculata*. Furthermore, quantification of three bioactive components was carried out using RRLC-TOF, which could provide useful information for quality control of *A. paniculata*. This study would facilitate the quality evaluation of *A. paniculata* for safe and efficacious use and be a powerful reference for the identification of similar compounds presented here by MS spectra.
